# Feasibility Study on Endoscopic Balloon-Assisted Laser Treatment (EBLT) of Gastroesophageal Reflux Disease (GERD) in In Vivo Porcine Model

**DOI:** 10.3390/biomedicines11061656

**Published:** 2023-06-07

**Authors:** Boram Cha, Hyejin Kim, Van Gia Truong, Sun-Ju Oh, Seok Jeong, Hyun Wook Kang

**Affiliations:** 1 Department of Internal Medicine, Inha University Hospital, Inha University College of Medicine, Incheon 22332, Republic of Korea; chaboram@hanmail.net; 2 TeCure, Inc., Busan 48548, Republic of Korea; hkim@tecure.co.kr (H.K.); gvtruong@tecure.co.kr (V.G.T.); 3 Department of Pathology, Kosin University College of Medicine, Busan 49267, Republic of Korea; hi-max@hanmail.net; 4 Department of Biomedical Engineering and MarineIntegrated Biomedical Technology Center, Pukyong National University, Busan 48513, Republic of Korea

**Keywords:** balloon-assisted laser treatment, catheter, endoscope, gastroesophageal reflux disease, porcine model

## Abstract

Gastroesophageal reflux disease (GERD) has been growing globally, with an increasing burden on the healthcare system due to multiple factors, such as aging and obesity. The current study evaluated the feasibility of endoscopic balloon-assisted laser treatment (EBLT) in a porcine model. GERD was initially developed in three animals via botulinum toxin injection into lower esophageal sphincter (LES). A week after the injection, the EBLT was performed on the GERD-developed models (control = 1 vs. treated = 2). A dose of 30 W of 980 nm laser light was endoscopically applied for 90 s to the LES. Both endoscopic ultrasound and manometry were performed before and after the EBLT. After 12 weeks, esophageal tissues were extracted and prepared for histological analysis. The maximum mucosa temperature was below 50 °C during the EBLT. Compared to control, the treated group yielded thicker and shorter LES muscle layers and maintained LES pressure. Through histology, the EBLT reinforced the muscularis layer with preserved mucosa and mild remodeling of the intermuscular collagen in the LES. The current study demonstrated the feasibility of EBLT as a new endoscopic approach for GERD. Further studies will examine the EBLT in a larger number of animals to warrant efficacy and safety for clinical translations

## 1. Introduction

Gastroesophageal reflux disease (GERD) is one of the most common chronic diseases in modern society caused by the reflux of stomach acid into the esophagus due to a weak or relaxed lower esophageal sphincter (LES) [1,2]. The global prevalence of GERD remains high and is increasing in the adult population. According to a meta-analysis published in the *Gut* journal in 2018, the prevalence of GERD in North America and Europe is estimated to be around 15–20% of the population, while the prevalence in Asia is estimated to be around 10% of the population [3]. In particular, the prevalence of doctor-diagnosed GERD in South Korea increased from 4.6% in 2005 to 7.3% in 2008 [4,5]. The common symptoms of GERD include chest pain, discomfort due to heartburn, regurgitation, and difficulty swallowing [2,6]. GERD can have a significant impact on a patient’s quality of life and can also increase the risk of developing complications, such as esophageal ulcer, stricture, erosion, and Barrett’s esophagus [1,2,6]. Treatments for GERD include lifestyle changes, such as avoiding trigger foods, losing weight, and quitting smoking, as well as medications, such as antacids, H2 blockers, and proton pump inhibitors (PPIs) [7,8]. The cost of drugs for PPIs was over USD 10 billion annually in the US alone [9]. Despite treatment with PPIs, about 30–40% of people with GERD continue to have persistent symptoms [7,8,10,11]. In addition, several studies have highlighted the potential adverse effects associated with prolonged use of PPIs (i.e., bone fractures, chronic kidney disease, and vitamin deficiencies) [1,2,6,9]. Thus, anti-reflux surgery may be needed to strengthen the LES or even to repair a hiatal hernia [7,10,11].

Endoscopic procedures have recently been introduced as an alternative to traditional laparoscopic fundoplication and the long-term use of medical drugs [12]. These techniques have become increasingly attractive to both patients and physicians as a potentially safe, effective, and minimally invasive procedure. The clinical goal is to reduce the frequency of reflux of stomach contents into the esophagus by reconstructing a sphincter that can have the reduced diameter of the LES. Three major endoscopic therapies have been FDA-approved and marketed in the US: EsophyX, MUSE, and Stretta. Both EsophyX (EndoGastric Solutions, Redmond, WA, USA) and MUSE (Medigus ultrasonic surgical endostapler; Medigus, Omer, Israel) are based on the transoral incisionless fundoplication (TIF) technique [13,14,15]. The EsophyX technique utilizes a unique suturing device to create a 3~5 cm anatomical valve that encloses around 200~300° [16,17,18]. The MUSE approach employs a flexible miniature camera, an ultrasonic probe, and a surgical stapler positioned at the tip of the endoscope to attach the upper portion of the stomach to the esophagus through a 270° anterior fundoplication [19,20]. Both the methods involve a partial wrap around the lower esophagus to create a mechanical valve at the esophagogatric junction that reduces reflux events. Lastly, the Stretta therapy (Restech, Houston, TX, USA) involves the application of temperature-controlled radiofrequency (RF) energy (65~85 °C) to the LES muscle layer for a few minutes via direct contact with multiple electrodes [19,21,22]. Thermal coagulation is induced to tighten and thicken the LES muscle, reducing the sensitivity of the esophagus to refluxed acid and other irritants and eventually preventing the reflux of stomach contents into the esophagus. However, these procedures still have limitations, such as being blind procedures, requiring general anesthesia, high level of technical skill, prolonged operation time, and risk of procedural complications [15]. Short-term complications include dysphagia, chest pain, sore throat, bleeding, and perforation, while long-term complications are related to recurrence, need for surgical revision, and erosive esophagitis [23,24,25,26]. Moreover, controversies remain in clinical practice regarding the durability of treatment, the capacity to normalize esophageal acid load in many patients, limited healing, and failure to improve LES pressure [15,24,25,27]. Many studies found that endoscopic therapies were more effective and less expensive than PPI medication after 10~30 years of follow-up [21,28,29]. These studies suggest that endoscopic strategies may be a cost-effective alternative to long-term PPI medication for the treatment of GERD [21,28,29]. However, the cost of endoscopic therapies is still high, at around USD 5000 to USD 10,000 per treatment session [21,29,30]. As a result, patients may be hesitant to undergo these procedures with potential risks when they can manage their symptoms with PPIs. Therefore, a new endoscopic device for the treatment of GERD still needs to be developed with enhanced safety, more favorable clinical outcomes, ease of use, and cost-effectiveness.

Endoscopic balloon-assisted laser treatment (EBLT) has been introduced for the endoscopic treatment of tubular tissues (e.g., biliary duct and esophagus) by inflating the balloon for tissue expansion and simultaneously irradiating the inner lumen with laser light for thermal ablation and/or coagulation [31,32,33,34]. A combination of mechanical expansion and cylindrical light emission during EBLT can selectively induce uniform coagulation in a target tissue with minimal thermal injury to the surrounding tissue in a short time (<10 min). The current study evaluated the feasibility of the EBLT for GERD in an in vivo porcine model. It was hypothesized that the non-contact cylindrical light emission during the EBLT could lead to the radial coagulation of the LES muscle, resulting in thicker muscularis mucosa and propria as a result of mild collagenous fibrosis, as well as no or minimal thermal injury to the mucosal surface. For the in vivo evaluations, GERD was developed in the animal models via injection of botulinum toxin into the LES. The GERD-developed animal models were used to assess the feasibility of the proposed EBLT for remodeling the LES muscle in terms of endoscope ultrasound, manometry, and histological analysis on the treated esophageal tissue.

## 2. Materials and Methods

### 2.1. Laser System and Device

**Figure 1:**
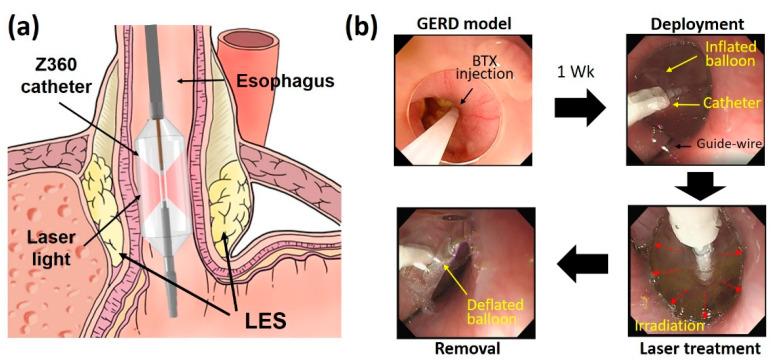
Schematic illustrations of endoscopic balloon-assisted laser treatment (EBLT) for GERD in in vivo porcine model: (**a**) position of catheter to irradiate LES and (**b**) steps of EBLT procedure (LES: lower esophageal sphincter; BTX: botulinum toxin).

The current study utilized a customized 30 W 980 nm laser system (EsoLight Z360, TeCure, Inc., Busan, Republic of Korea) in continuous wave mode for laser treatment. A balloon-assisted optical catheter was used to deliver the laser light endoscopically and to perform the EBLT of GERD in in vivo porcine models, as shown in Figure 1a. The catheter consisted of a transparent balloon (diameter = 22 mm), a diffusing applicator (element = 10 mm), and a temperature sensor for real-time monitoring during the EBLT.

### 2.2. In Vivo Tests

For the current feasibility study, three male pigs (40 ± 8 kg) were tested for the EBLT of GERD in in vivo models. The animals were obtained from CRONEX Inc. (Cheongju, Republic of Korea) and housed in individual cages under adequate temperature and humidity conditions. After acclimation for a week, all the animals were divided into two groups for comparison: control group (CTRL; *n* = 1) and treated group (*n* = 2). Before the experiments, the animals were fasted for 48 h and then anesthetized with 0.1 mL/kg mixture of zoletil and xylazine (1:1 ratio). During the experiments, the general anesthesia was provided by a mechanical ventilator with isoflurane (1~2%) and oxygen (2 L/min). Under the anesthesia, the baseline of the esophagogastric (EG) junction was firstly confirmed through an endoscope. Afterwards, botulinum toxin donated from Daewoong Pharmaceutical Co., Ltd. (BTX; Seoul, Republic of Korea) was injected into the muscle layer at the EG junction (25 units per quadrant), and the animals were kept for a week to weaken the LES muscle layer to fabricate a GERD model [35]. After the development of the GERD model, a catheter was inserted along a guide-wire into the esophagus of each animal under the endoscopy, and the catheter tip was positioned at the EG junction (Figure 1a). The balloon of the catheter was then inflated with cold balloon fill media. For the EBLT, laser energy of 2700 J (30 W for 90 s) was delivered to three positions at the EG junction (i.e., −2, 0, and 2 cm away from EG junction). The control group underwent a sham operation at the same positions. During the EBLT, mucosa temperature was monitored in real-time mode to spare the mucosa from thermal injury and to ensure treatment safety. After the EBLT, the balloon was deflated, and the catheter was removed (Figure 1b). All the tested animals were kept alive for 12 weeks to evaluate the chronic responses of the esophageal tissues. Body weight measurements and blood tests were conducted pre- and post-BTX injection every four weeks after the EBLT. All the in vivo experiments were conducted in accordance with the standard guidelines of the Korean National Institutes of Health (KNIH) guidelines for the care and use of laboratories. The current experiments were approved by the Institutional Animal Care and Use Committee of KNOTUS, Korea (permit number: KNOTUS IACUC 22-KE-0285).

### 2.3. Endoscopic Ultrasound (EUS) Monitoring and Manometry

Before and after in vivo tests, all animals underwent endoscopic ultrasound (EUS) monitoring and manometry under anesthesia. A GF-UCT260 linear-array echo endoscope (Olympus Medical Co. Ltd., Tokyo, Japan) was employed with an EU-ME2 ultrasound system (Olympus Medical Co. Ltd., Tokyo, Japan) to measure the thickness of an LES muscle layer in the porcine esophageal tissue. The captured images were analyzed with Image J (National Institute of Health, Bethesda, MD, USA) to compare the measured thicknesses between control and treated groups before and after EBLT. Esophageal manometry (ManoScan, Medtronic, MN, USA) was performed to determine both LES length and pressure pre- and post-EBLT. All the recorded data were assessed using analysis software (ManoView ESO, Medtronic, MN, USA). All the measured LES pressures were normalized by the initial pressure for comparison

### 2.4. Histology Analysis

Twelve weeks after EBLT, all the tested animals were euthanized by intravenous administration of 100 mg/kg pentobarbital for histological analysis. Thereafter, all esophageal tissues were extracted from the animals, and each specimen (±2 cm from EG junction) was fixed in 10% neutral formalin solution for four days. The fixed tissues were sectioned in a thickness of 4 µm to prepare histology slides. Each slide was stained with both hematoxylin and eosin (HE) and Masson’s trichrome (MT) under the standard protocol. All the stained slides were scanned using a digital slide scanner (Motic Easy Scan Pro Digital Slide Scanner, Motic Asia Corp., Kowloon, Hong Kong) and observed with DSAssistant software to analyze biological and structural changes in the tissues. Then, a pathologist examined all the slides and scored the histological responses of the random tissue regions semi-quantitatively by grading both inflammation and fibrosis on a scale ranging from 0 to 4 (i.e., 0 = none, 1 = minimal, 2 = mild, 3 = moderate, and 4 = severe). The thicknesses of muscularis mucosa (MM) and muscularis propria (MP) were measured using the DSAssistant software for quantitative comparison.

## 3. Results

**Figure 2:**
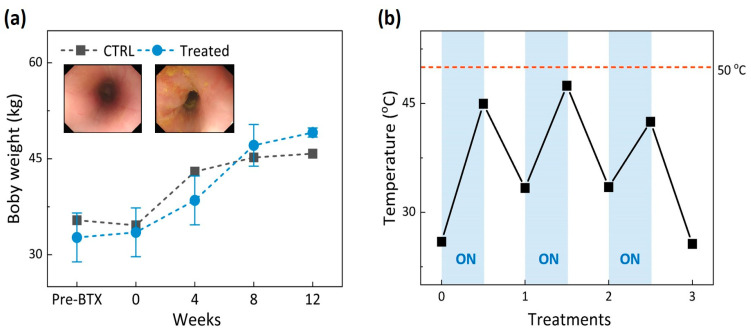
Evaluations of porcine model after EBLT: (**a**) body weight (every four weeks) and (**b**) real-time monitoring of mucosa temperature during EBLT. Blue areas in (**b**) represent laser irradiation time.

Figure 2a demonstrates that the body weights of the animals increased over time up to 12 weeks after EBLT for both control and treated groups. Blood tests confirmed that all the measured values remained in normal ranges post-EBLT (Table 1). The endoscopic images in Figure 2a evidenced no irreversible thermal injury (discoloration or denaturation) on the treated esophageal mucosa. Figure 2b shows the temporal developments of mucosa temperature during three rounds of EBLT. It was noted that the maximum temperature during laser irradiation merely reached 47 °C, indicating that the mucosa experienced mild hyperthermia and the mucosa surface was preserved safely during the EBLT.

**Figure 3:**
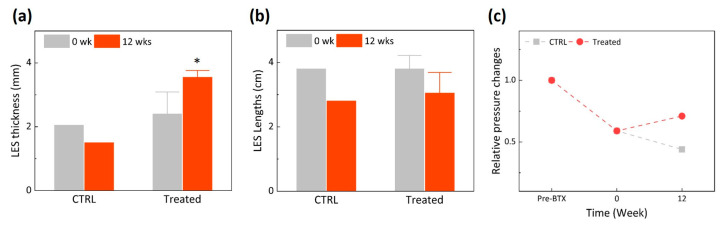
Quantitative evaluations of lower esophageal sphincter (LES) before and after EBLT: (**a**) LES thickness, (**b**) LES length, and (**c**) relative changes in LES pressure (* *p* < 0.05).

Figure 3 presents physical changes in thickness, length, and pressure of LES 12 weeks after EBLT. According to Figure 3a, the LES thickness from the treated group increased by up to 50% (from 2.4 to 3.6 mm) after the EBLT, whereas that from the control group decreased by 27% (from 2.1 to 1.5 mm). It was confirmed that the initial LES thicknesses from both groups (0 week) were comparable. Regardless of groups, LES lengths remained almost unvaried between pre- and post-BTX injection (Figure 3b). The LES length from the control group decreased by 7% more than that from the treated group (26% for control vs. 19% for treated; Figure 3b). According to Figure 3c, the LES pressure significantly dropped in both the control and treated groups after BTX injection as BTX-induced LES became weakened [35]. Although the control group showed a continuous decrease in the LES pressure, the treated group increased the LES pressure 12 weeks after the EBLT.

**Figure 4:**
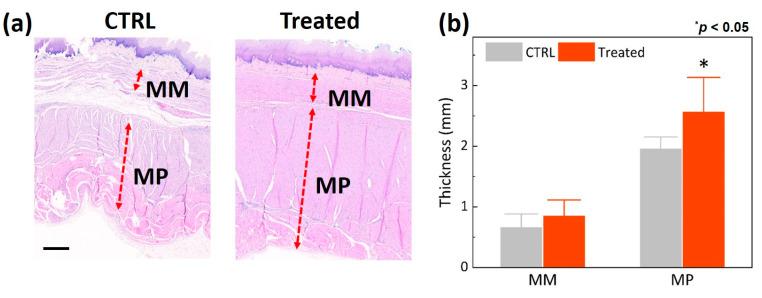
Histological analysis of LES muscle layer in porcine esophagus 12 weeks after EBLT: (**a**) HE-stained images (scale bar = 500 µm) and (**b**) comparison of muscularis mucosa (MM) and muscularis propria (MP).

**Figure 5:**
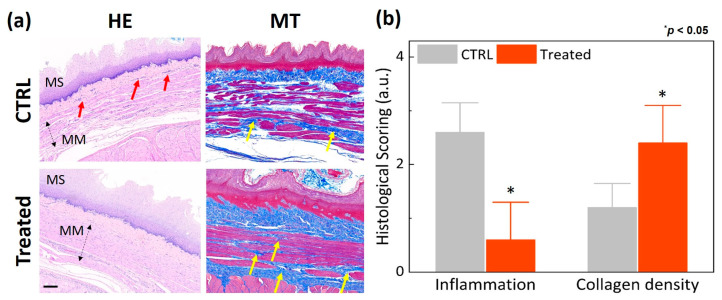
Semi-quantitative analysis of porcine esophagus 12 weeks after EBLT: (**a**) HE- and MT-stained images (scale bar = 200 µm) and (**b**) comparison of inflammation and collagen density. Red and yellow arrows represent inflammatory cells and collagen in muscularis mucosa, lamina propria, and submucosa, respectively. Note that red and blue colors in the MT images mean muscle and collagen, respectively (MS: mucosa; MM: muscularis mucosa).

Figure 4 shows the histologic analysis conducted to evaluate morphological changes at the EG junction 12 weeks after EBLT. According to Figure 4a, the treated group yielded thicker MM and MP along with dense collagen orientation compared to the control group. No significant thermal injury was found in the mucosa layer of the treated esophagus surface (right image in Figure 4a). Figure 4b demonstrates that the treated group yielded 30% and 31% thicker MM and MP, respectively, than the control group (MM = 654 μm for control vs. 847 μm for treated; MP = 1952 μm for control vs. 2561 μm for treated; *p* < 0.05). Figure 5 exhibits semi-quantitative evaluations of the degree of inflammation and collagen promotion in the esophagus tissues after the EBLT. According to Figure 5a, the treated group presented no or minimal inflammatory cells in the lamina propria (HE) and a noticeable promotion of collagen density (MT) in the muscularis mucosa, lamina propria, and submucosa layers, which resulted in thickening the LES muscle. Figure 5b demonstrates that compared to the control group, the treated group yielded 77% less inflammatory responses but 50% higher collagen density. The histological evaluations confirmed that the proposed EBLT was able to selectively thicken the LES muscle layer via collagen remodeling (synthesis, production, and tightening) with minimal inflammation and no mucosa damage.

## 4. Discussion

The goal of the current study was to assess the feasibility of EBLT for GERD in the in vivo disease-developed porcine model. Experimental findings confirmed that the EBLT was able to thicken the LES muscle layer and to increase the LES pressure without damage to the mucosa (Figure 3, Figure 4 and Figure 5). The mechanism of action can be that endoscopic and circumferential laser irradiation from a balloon-assisted optical catheter entails the coagulation of the radial tissue of the LES muscle, eventually reinforcing MM and MP via collagen remodeling and increasing the resting pressure of the LES via loss of tissue compliance. Similarly, previous studies on the Stretta treatment reported that the application of RF energy causes thermal coagulation in esophageal tissue, leading to the restoration of the LES pressure and a reduction in transient lower esophageal sphincter relaxations (TLESRs). In spite of favorable clinical outcomes, the Stretta treatment is still associated with a lengthy procedure time (~1 h) due to the multiple insertions of RF needle electrodes (10~15 times) into the esophagus tissue [10]. In addition, as a large-sized catheter (~1 cm in diameter) prevents the concurrent use of an endoscope, a blind procedure is performed during the Stretta treatment, which can be associated with difficult operation, low treatment precision, and less effectiveness. On the other hand, the current EBLT uses a small-sized catheter (~3 mm in diameter) that can be used concomitantly with the endoscope, which can help to monitor the target area during the treatment and improves treatment efficacy. The non-contact application of laser light can cover a large surface of the targeted esophageal tissue, significantly shortening the entire procedure time (within 10~15 min) and preserving the mucosa surface during the treatment (no thermal injury). Therefore, in order to confirm the potential clinical benefits, further studies will directly compare the proposed EBLT to the Stretta procedure for GERD in terms of treatment efficacy/safety, procedural convenience, operation time, and technical success.

The current findings demonstrated no thermal injury to the mucosa surface after EBLT, as the mucosa temperature was maintained below 50 °C (Figure 2). It was noted that the temperature increased only during laser irradiation (Figure 2b). The comparable changes in body weight were noticed for the control and treated groups (Figure 2a), implying that the mucosa and treated lesion in the muscle layer hardly affected the intake and digestion of food. On the other hand, morphological changes were found markedly in the LES muscle layer as a result of selective thermal coagulation after the EBLT (Figure 4 and Figure 5). As cold balloon fill media (~10 °C) was used to inflate the balloon, the initial temperature of the mucosa surface became lower (~27 °C) than the body temperature (37 °C). In spite of light absorption during the irradiation, the mucosa experienced a slow increase in temperature, which barely reached 50 °C and was able to preserve the mucosa surface during the EBLT. Instead, the muscle layer below the mucosa could have undergone a relatively rapid temperature elevation, which accompanied the eventual thermal coagulation in the LES according to Truong et al. [39]. To elucidate a spatio-temporal distribution of temperature fields in the tissue during the EBLT, multiple real-time temperature measurements will thereby be conducted in both the mucosa and the muscle (2~3 mm below mucosa) simultaneously. Additionally, the treated group showed an increase in relative LES pressure after the EBLT, whereas the control group was associated with a continuous decrease in LES pressure. The current trend shows a good agreement with the Stretta study in terms of the increase in the LES pressure [35,40]. However, more data points should be collected for statistical comparison to confirm the potential benefits of the EBLT. Furthermore, not only the LES pressure but also gastric pressure should be measured together to validate the augmentation of the LES function and the neuromodulation of TLESRs for clinical outcomes of reflux reduction [35,40,41,42]. In addition, to elucidate the mechanism of the EBLT on the muscle layer, further histopathological evaluations, such as immunohistochemistry, will be conducted to examine the activation of resident fibroblasts and inflammatory cells post-EBLT [43].

Although the current study demonstrated the feasibility of EBLT for GERD in an in vivo model, experimental limitations still remain. A small number of animals was used for EBLT testing because of the required initial development of GERD in a porcine model and experimental validation of the proposed EBLT. Therefore, a survival study with a large number of animals should be conducted to statistically assess both the efficacy and long-term safety of the proposed method [44]. The current EBLT targets LES to increase the thickness and the resting pressure of the LES, eventually aiming to minimize GERD-related symptoms. Recently, it was reported that treatments (coagulation or incision) on gastric cardia consisting of sling and clasp fibers could entail less tissue compliance, reducing TLESRs and improving reflux symptoms [35,40,41,42]. Thus, laser energy should be applied to the gastric cardia to identify whether the efficacy of the EBLT can be maximized by reinforcing the sling fibers at the EG junction via collagen remodeling and altering vagal neuromodulation of the TLESRs. As the current study applied a single condition for the EBLT, multiple parameters, such as laser power, irradiation time, and treatment length, should be examined and optimized to achieve favorable clinical outcomes. Hence, further studies will evaluate the proposed EBLT in a large number of GERD-developed animal models in terms of treatment dosimetry, acute/chronic wound healing, and reduction in GERD-related symptoms prior to clinical translations.

## 5. Conclusions

The current study demonstrated the feasibility of EBLT in an in vivo porcine model with GERD. Both non-contact circumferential laser irradiation and real-time temperature monitoring helped to increase LES thickness and pressure and to preserve mucosa surface without thermal injury. Further studies will investigate the proposed EBLT of GERD in a larger number of in vivo models in order to elucidate the mechanism of action for functional and structural changes in LES and to warrant efficacy and safety for clinical translations.

## Figures and Tables

**Table 1:** Comparison of blood contents after EBLT in porcine model [36,37,38].

	Normal Range	CTRL					Treated				
		Pre-BTX	Post-BTX	4 w	8 w	12 w	Pre-BTX	Post-BTX	4 w	8 w	12 w
WBC (×10^3^ cells/µL)	13.6–32.2	16.9	14.97	25.86	26.56	18.4	26.24	22.01	24.59	19.81	12.61
Neutrophil (%)	8–56.9	36.7	30.2	43.5	40	56.4	44.2	40.4	30.25	23.65	32.25
Eosionophil (%)	0–6	0.2	1.4	1.7	2.2	0.2	0.15	0.5	1.1	1.5	0.15
Lymphocyte (%)	20.9–74	56.6	62.1	50.4	42.2	41	50.35	53.65	64.45	69.7	63.3
meanHb (g/dL)	10.8–14.8	12.6	10.9	12.9	14	12.2	13.25	11.8	14.05	14.65	12.1
Plt (×10^3^ cells/µL)	155–686	383	264	415	679	337	582.5	434	378	366	355

## Data Availability

Data will be made available on request.

## References

[1] Clarrett D.M., Hachem C. (2018). Gastroesophageal reflux disease (GERD). Mo. Med..

[2] Fass R., Boeckxstaens G.E., El-Serag H., Rosen R., Sifrim D., Vaezi M.F. (2021). Gastro-oesophageal reflux disease. Nat. Rev. Dis. Prim..

[3] Eusebi L.H., Ratnakumaran R., Yuan Y., Solaymani-Dodaran M., Bazzoli F., Ford A.C. (2018). Global prevalence of, and risk factors for, gastro-oesophageal reflux symptoms: A meta-analysis. Gut.

[4] Kim K.M., Cho Y.K., Bae S.J., Kim D.S., Shim K.N., Kim J.H., Jung S.W., Kim N. (2012). Prevalence of gastroesophageal reflux disease in Korea and associated health-care utilization: A national population-based study. J. Gastroenterol. Hepatol..

[5] Min B.-H., Huh K.C., Jung H.-K., Yoon Y.H., Choi K.D., Song K.H., Keum B., Kim J.W. (2014). Prevalence of uninvestigated dyspepsia and gastroesophageal reflux disease in Korea: A population-based study using the Rome III criteria. Dig. Dis. Sci..

[6] Kahrilas P.J. Complications of Gastroesophageal Reflux in Adults. https://www.medilib.ir/uptodate/show/2263.

[7] Sandhu D.S., Fass R. (2018). Current trends in the management of gastroesophageal reflux disease. Gut Liver.

[8] Kroch D.A., Madanick R.D. (2017). Medical treatment of gastroesophageal reflux disease. World J. Surg..

[9] Jaynes M., Kumar A.B. (2019). The risks of long-term use of proton pump inhibitors: A critical review. Ther. Adv. Drug Saf..

[10] Triadafilopoulos G. (2014). Stretta: A valuable endoscopic treatment modality for gastroesophageal reflux disease. World J. Gastroenterol. WJG.

[11] Chang P., Friedenberg F. (2014). Obesity and GERD. Gastroenterol. Clin..

[12] Muthusamy V.R., Lightdale J.R., Acosta R.D., Chandrasekhara V., Chathadi K.V., Eloubeidi M.A., Fanelli R.D., Fonkalsrud L., Faulx A.L., Khashab M.A. (2015). The role of endoscopy in the management of GERD. Gastrointest. Endosc..

[13] Flores L., Krause C., Pokala B., Hosein S., Armijo P.R., Mishra T., Kothari S., Oleynikov D. (2019). Novel therapies for gastroesophageal reflux disease. Curr. Probl. Surg..

[14] Lee D.P., Chang K.J. (2022). Endoscopic Management of GERD. Dig. Dis. Sci..

[15] Shibli F., Fass R. (2021). Endoscopic Anti-Reflux Procedures: Ready for Clinical Use?. Curr. Treat. Options Gastroenterol..

[16] Fass R. (2017). An overview of transoral incisionless fundoplication and magnetic sphincter augmentation for GERD. Gastroenterol. Hepatol..

[17] Chang K.J., Bell R. (2020). Transoral incisionless fundoplication. Gastrointest. Endosc. Clin..

[18] Ihde G.M. (2020). The evolution of TIF: Transoral incisionless fundoplication. Ther. Adv. Gastroenterol..

[19] Fass R. (2019). Endoscopic approaches for the treatment of gastroesophageal reflux disease. Gastroenterol. Hepatol..

[20] Zacherl J., Roy-Shapira A., Bonavina L., Bapaye A., Kiesslich R., Schoppmann S.F., Kessler W.R., Selzer D.J., Broderick R.C., Lehman G.A. (2015). Endoscopic anterior fundoplication with the Medigus Ultrasonic Surgical Endostapler (MUSE™) for gastroesophageal reflux disease: 6-month results from a multi-center prospective trial. Surg. Endosc..

[21] Sandhu D.S., Fass R. (2019). Stretta therapy in the management of gastroesophageal reflux disease (GERD). Ann Esophagus.

[22] Sowa P., Samarasena J.B. (2020). Nonablative radiofrequency treatment for gastroesophageal reflux disease (STRETTA). Gastrointest. Endosc. Clin..

[23] Richter J.E. (2013). Gastroesophageal reflux disease treatment: Side effects and complications of fundoplication. Clin. Gastroenterol. Hepatol..

[24] Huang X., Chen S., Zhao H., Zeng X., Lian J., Tseng Y., Chen J. (2017). Efficacy of transoral incisionless fundoplication (TIF) for the treatment of GERD: A systematic review with meta-analysis. Surg. Endosc..

[25] Fass R., Cahn F., Scotti D.J., Gregory D.A. (2017). Systematic review and meta-analysis of controlled and prospective cohort efficacy studies of endoscopic radiofrequency for treatment of gastroesophageal reflux disease. Surg. Endosc..

[26] Zerbib F., Sacher-Huvelin S., Coron E., Coffin B., Melchior C., Ponchon T., Cholet F., Chabrun E., Vavasseur F., Gorbatchef C. (2020). Randomised clinical trial: Oesophageal radiofrequency energy delivery versus sham for PPI-refractory heartburn. Aliment. Pharmacol. Ther..

[27] Lipka S., Kumar A., Richter J.E. (2015). No evidence for efficacy of radiofrequency ablation for treatment of gastroesophageal reflux disease: A systematic review and meta-analysis. Clin. Gastroenterol. Hepatol..

[28] Park S., Park S., Park J.-M., Ryu S., Hwang J., Kwon J.-W., Seo K.W. (2020). Anti-reflux surgery versus proton pump inhibitors for severe gastroesophageal reflux disease: A cost-effectiveness study in Korea. J. Neurogastroenterol. Motil..

[29] Funk L.M., Zhang J.Y., Drosdeck J.M., Melvin W.S., Walker J.P., Perry K.A. (2015). Long-term cost-effectiveness of medical, endoscopic and surgical management of gastroesophageal reflux disease. Surgery.

[30] Thosani N., Goodman A., Manfredi M., Navaneethan U., Parsi M.A., Smith Z.L., Sullivan S.A., Banerjee S., Maple J.T. (2017). Endoscopic anti-reflux devices (with videos). Gastrointest. Endosc..

[31] Jeong S., Bak J., Kim S.M., Kang H.W. (2020). Feasibility study of endoscopic thermal coagulation with circumferential laser irradiation for treating esophageal tissue. Lasers Med. Sci..

[32] Bak J., Hwang J., Park S., Kang H.W. (2017). Integration of optical applicator with balloon catheter for photothermal treatment of biliary stricture. Lasers Surg. Med..

[33] Park J.-S., Jeong S., Lee D.H., Kim J.M., Kim S.M., Kang H.W. (2021). The use of a 532-nm laser fitted with a balloon and a cylindrical light diffuser to treat benign biliary stricture: A pilot study. Lasers Med. Sci..

[34] Truong V.G., Jeong S., Lee Y., Kim S.M., Kang H.W. Novel endoscopic laser treatment of common bile duct stenosis using balloon catheter-integrated diffusing applicator (BCDA). Proceedings of the SPIE Advanced Biophotonics Conference (SPIE ABC 2021).

[35] Utley D.S., Kim M., Vierra M.A., Triadafilopoulos G. (2000). Augmentation of lower esophageal sphincter pressure and gastric yield pressure after radiofrequency energy delivery to the gastroesophageal junction: A porcine model. Gastrointest. Endosc..

[36] Lim S., Truong V.G., Choi J., Jeong H.J., Oh S.-J., Park J.-S., Kang H.W. (2022). Endoscopic ultrasound-guided laser ablation using a diffusing applicator for locally advanced pancreatic cancer treatment. Cancers.

[37] Sipos W., Duvigneau C.J., Hartl R.T., Schwendenwein I. (2011). Exploratory reference intervals on hematology and cellular immune system of multiparous Large White sows. Vet. Immunol. Immunopathol..

[38] Ježek J., Starič J., Nemec M., Plut J., Oven I.G., Klinkon M., Štukelj M. (2018). The influence of age, farm, and physiological status on pig hematological profiles. J. Swine Health Prod..

[39] Truong V.G., Kim H., Lee B.-I., Cha B., Jeong S., Oh S.-J., Kang H.W. (2023). Development of Novel Balloon-Integrated Optical Catheter for Endoscopic and Circumferential Laser Application. Ann. Biomed. Eng..

[40] Herman R.M., Berho M., Murawski M., Nowakowski M., Ryś J., Schwarz T., Wojtysiak D., Wexner S.D. (2015). Defining the histopathological changes induced by nonablative radiofrequency treatment of faecal incontinence–a blinded assessment in an animal model. Color. Dis..

[41] Kim M.S., Holloway R.H., Dent J., Utley D.S. (2003). Radiofrequency energy delivery to the gastric cardia inhibits triggering of transient lower esophageal sphincter relaxation and gastroesophageal reflux in dogs. Gastrointest. Endosc..

[42] Ayazi S., Tamhankar A., DeMeester S.R., Zehetner J., Wu C., Lipham J.C., Hagen J.A., DeMeester T.R. (2010). The impact of gastric distension on the lower esophageal sphincter and its exposure to acid gastric juice. Ann. Surg..

[43] Rieder F., Biancani P., Harnett K., Yerian L., Falk G.W. (2010). Inflammatory mediators in gastroesophageal reflux disease: Impact on esophageal motility, fibrosis, and carcinogenesis. Am. J. Physiol.-Gastrointest. Liver Physiol..

[44] Noar M., Squires P., Noar E., Lee M. (2014). Long-term maintenance effect of radiofrequency energy delivery for refractory GERD: A decade later. Surg. Endosc..

